# Risk factors impacting intra- and postoperative cerebrospinal fluid rhinorrhea on the endoscopic treatment of pituitary adenomas: A retrospective study of 250 patients

**DOI:** 10.1097/MD.0000000000027781

**Published:** 2021-12-10

**Authors:** Ming Wang, Yang Cai, Yugang Jiang, Yong Peng

**Affiliations:** Department of Neurosurgery, the Second Xiangya Hospital of Central South University, Changsha, Hunan, China.

**Keywords:** cerebrospinal fluid leak, endoscopic transsphenoidal surgery, pituitary adenoma, risk factors

## Abstract

We aimed to identify the risk factors associated with intra- and postoperative cerebrospinal fluid (CSF) leakage in pituitary adenomas treated with endoscopic transsphenoidal surgery.

This study is a retrospective analysis of 250 pituitary adenoma cases from January 2017 to December 2019 at our hospital. All patients underwent endoscopic endonasal transsphenoidal surgeries. Univariate and multivariate analyses were performed to investigate the risk factors associated with intra- and postoperative CSF rhinorrhea.

Eighty (32.0%) and nine (3.6%) patients had intra- and postoperative CSF leakage, respectively. Tumor size was an independent risk factor for intraoperative CSF leakage (odds ratio [OR], 1.229; 95% confidence interval [CI], 1.133–1.334; *P* < .001); intraoperative CSF leakage was an independent risk factor for postoperative CSF leakage (OR, 7.707; 95% CI, 1.336–44.455; *P* = .022). Chronic respiratory disease (OR, 57.500; 95% CI, 8.031–411.682; *P* < .001) was also an independent risk factor for postoperative CSF leakage. Vascularized septal mucosal flap was a protective factor (OR, 0.107; 95% CI, 0.013–0.894; *P* = .039).

Intraoperative CSF leakage is more likely to occur in large pituitary adenomas. In the presence of intraoperative CSF leakage, postoperative CSF rhinorrhea is very likely to occur. Patients with chronic respiratory disease are also more likely to develop postoperative CSF leakage. The sellar base reconstructed using vascularized nasal septal flaps can significantly decrease the risk. The Knosp grade, degree of tumor resection, and postoperative use of a lumbar subarachnoid drain did not have any effects on postoperative CSF rhinorrhea.

## 1 Introduction

Pituitary adenomas account for 15% of all intracranial tumors.^[1]^ Endoscopic endonasal transsphenoidal surgery (EETS) has become the standard surgical treatment for pituitary adenomas. It is a reasonably safe procedure with low morbidity, particularly when performed by experienced surgeons.^[2–4]^ However, postoperative cerebrospinal fluid (CSF) leakage is a major life-threatening complication following EETS,^[3]^ which may lead to meningitis^[5]^ or tension pneumocephalus,^[6]^ and is associated with prolonged hospitalization. The incidence of such complications ranges from 0.5 to 15% following EETS.^[3]^ Identifying the risk factors that affect the incidence of intra- and postoperative CSF leakage may help in decreasing the rates of these complications. In the present study, we retrospectively reviewed 375 serial cases of pituitary adenomas treated with EETS by a single surgeon. Our primary aim was to identify the predictors of intra- and postoperative CSF leakage in patients who underwent endoscopic resection of pituitary adenomas to better inform patients and educate surgeons on this potential complication.

## 2 Methods

### 2.1 Patient population and data collection

A retrospective review of 315 consecutive patients with pituitary adenomas from January 2017 to December 2019 was conducted. All patients were followed up for 1 to 3 years, with an average follow-up period of 15.6 months. One surgeon (Y.J.) performed the initial EETS for all patients. This study was approved by the Ethics Committee of our hospital (approval number:2020080). The inclusion criteria were as follows: 1) a histological diagnosis of pituitary adenoma, 2) primary tumor, 3) complete resection of the tumor by EETS, 4) complete surgery performed by Y.J., and 5) complete medical records available. Patients with incomplete data, tumor recurrence, prior skull-base operation, or a history of radiation therapy or chemotherapy, were excluded from this study.

The following data were collected retrospectively from the medical records: demographic data, including age, race, sex, and body mass index (BMI), comorbidity, patient history, and perioperative data including hypertension, diabetes mellitus, coronary heart disease, chronic pulmonary disease, and preoperative administration of bromocriptine. Furthermore, information regarding tumor characteristics including tumor size, consistency of the adenoma, adenoma pathology type, Knosp grade, hemorrhage into adenoma, and extent of tumor excision were also documented. Other recorded features included reconstructive techniques and the use of CSF diversion. The primary outcome was intra- and postoperative CSF leakage.

### 2.2 Surgical procedure and skull base reconstruction

All patients underwent EETS for tumor resection. After infiltrating the local nasal mucosa with lidocaine and epinephrine, we commenced with bilateral middle turbinate reduction, and a vascularized nasal septal flap (VNSF) was then created for the large adenomas (≥10 mm). Diamond burs were used to shorten the posterior septum, and a sphenoidotomy was performed using a microdebrider until the back wall of the sphenoid and the borders of the sella turcica (sella) were visualized. The base of the sella range was located by a neuronavigator, and then opened using a high-speed drill and enlarged using rongeurs. A cruciate dural incision was created. Subsequently, the tumor was resected using suction and ring curettes within the pseudocapsule of the pituitary adenoma. To detect occult intraoperative CSF leakage before closure, we occasionally used the Valsalva maneuver.

In the absence of CSF leakage during EETS, we performed either no packing or packing with absorbable hemostatic gauze (Surgicel) within the sella, covered by one layer of synthetic dura, followed by a layer of fibrin glue or VNSF (for large adenomas). If there is evidence of minor intraoperative CSF leakage, we utilized fat graft packing of the sella and carefully reconstructed the sella floor with one layer of synthetic dura + VNSF or a free graft harvested from the nasal mucosa (for microadenomas). The surface of the above three repair materials was usually fixed with fibrin glue. In cases of major CSF leakage, the same procedures as a minor CSF leakage repair was followed, with the addition of an immediate lumbar CSF diversion for 5 to 7 days. The sella was supported by a Foley balloon catheter (12–14 French) for 5 to 7 days.

### 2.3 Diagnosis and management of cerebrospinal fluid leakage

The diagnosis of CSF leakage was made as follows. (I) For intraoperative CSF leak diagnosis: (1) intrasellar and/or parasellar outflow of a clear fluid, and (2) Valsalva maneuver as necessary. (II) For postoperative CSF leak diagnosis: (1) clinical nasal outflow and positive glucose outflow, and (2) nasal endoscopic examination as necessary. Postoperatively, patients with minor and intermittent CSF leakages were treated with bedrest (with the patient's head raised by 30 degrees), prophylactic antibiotics, and limited activities. Patients with major and persistent CSF leakages were treated with CSF diversion and lumbar drains. A lumbar drain was also performed for patients who did not improve after 7 days of bedrest. All patients who did not improve after CSF diversion treatment (≥7 days) were reoperated on via EETS.

### 2.4 Statistical methods

Categorical variables are presented as numbers and percentages and compared using the chi-squared test and Fisher exact test. Continuous variables are presented as means and standard deviations and compared using the two-sided unpaired *t*-test. Factors predictive of CSF leakage in the univariate analysis (*P* < .05) were entered into a stepwise binary logistic regression model to identify independent factors for intra- and postoperative CSF leakages. Statistical analysis was performed using SPSS (version 22.0; IBM Corporation, Armonk, NY). Statistical significance was set at *P* < .05.

## 3 Results

### 3.1 Patient characteristics

Of the 315 consecutive patients diagnosed with pituitary adenomas, a total of 250 patients met the inclusion criteria and were included in this study from January 2017 to December 2019 at our institution. The average age of the patients was 44.1 ± 12.3 years. The mean hospitalization time was 8.4 days. The pathology included 187 (74.8%) non-functioning adenomas, 40 (16.0%) GH-secreting pituitary adenomas, 3 (1.2%) PRL-secreting pituitary adenomas, and 20 (8.0%) ACTH-secreting pituitary adenomas. There were 30 (12.0%) cases of microadenomas, 205 (82.0%) cases of macroadenomas, and 15 (6.0%) cases of giant adenomas.

### 3.2 Intra- and postoperative CSF leakages

Intraoperative CSF leakages were observed during surgery in 80 patients (32.0%), of the 80 cases with intraoperative leaks, 61 were minor CSF leaks. Postoperative CSF leaks occurred in nine patients (3.6%), including seven patients with intraoperative CSF leaks and two patients without any recognized intraoperative CSF leakage. Among the nine patients with postoperative CSF rhinorrhea, six patients were initially treated with 5 to 7 days of passive lumbar drainage, including bedrest, prophylactic antibiotics, and limited activities. The three patients required surgical repair via a trans-sphenoidal approach. The CSF leakage in all nine patients resolved. Four patients with postoperative CSF leaks were complicated with meningitis 2 to 7 days after EETS. Those patient received antibiotics (meropenem + vancomycin) for 14 days and were discharged from the hospital without any complications.

### 3.3 Univariate analysis

In the univariate analysis, there were three factors associated with an increased intraoperative CSF leakage rate (Table 1): (1) BMI (*P* = .013), (2) tumor size (*P* < .001), and (3) consistency of the adenoma (*P* = .032). Similarly, predictors of postoperative CSF leaks according to the univariate analysis were (Table 3): (1) chronic respiratory disease (CRD) (*P* < .001), (2) degree of tumor resection (*P* = .011), and (3) intraoperative CSF leakage (*P* = .021). Only one factor was associated with a decreased postoperative CSF leakage rate, namely, the sella reconstructed by abdominal fascial graft + synthetic dura + VNSF (*P* < .001).

**Table 1:** Univariate analysis of impact of clinical characteristics associated with intraoperative CSF leak.

Variable	No intraop leak (n = 170)	Intraop leak (n = 80)	*P* value
Gender			
Female	82 (48.2%)	48 (60.0%)	.219
Male	88 (51.8%)	32 (40.0%)	
Age (yr)	44.15 ± 14.52	44.13 ± 15.85	.992
BMI (kg/m^2^)			
<18.5	24 (14.1%)	20 (25.0%)	.013
18.5–23.9	88 (51.8%)	50 (62.5%)	
24–27.9	44 (25.9%)	6 (7.5%)	
≥2 8.0	14 (8.2%)	4 (5.0%)	
Hypertension			
No	144 (84.7%)	60 (75.0%)	.191
Yes	26 (15.3%)	20 (25.0%)	
Diabetes			
No	154 (90.6%)	72 (90.0%)	1.000
Yes	16 (9.4%)	8 (10.0%)	
CRD			
No	162 (95.3%)	72 (95.0%)	1.000
Yes	8 (4.7%)	4 (5.0%)	
Coronary heart disease			
No	154 (90.6%)	76 (95.0%)	.621
Yes	16 (9.4%)	4 (5.0%)	
Knosp Grade			
0	28 (16.5%)	16 (20.0%)	.641
1–2	106 (62.4%)	40 (50.0%)	
3–4	36 (21.2%)	24 (30.0%)	
Tumor size	16.84 ± 6.16	26.20 ± 7.83	<.001
Consistency of the adenoma			.032
Tenacious	50 (29.4%)	57 (71.3%)	
Soft	120 (70.6%)	23 (28.7%)	
Degree of tumor resection			
GTR	152 (89%)	66 (83%)	.248
STR	14 (8.2%)	12 (15%)	
PR	4 (2.8%)	2 (2%)	
Pathology			
NF	124 (72.9%)	63 (78.8%)	.972
ACTH	16 (9.4%)	4 (5.0%)	
GH	28 (16.5%)	12 (15%)	
PRL	2 (1.2%)	1 (1.2%)	

**Table 2:** Multivariate analysis of impact of clinical characteristics upon intraoperative CSF leak.

Variable	OR	95% CI for OR	*P* value
Tumor size	1.229	1.133–1.334	<.001

**Table 3:** Univariate analysis of impact of clinical characteristics associated with postoperative CSF leak.

Variable	No postop leak (n = 241)	postop leak (n = 9)	*P* value
Gender			
Female	129 (53.5%)	5 (55.6%)	1.000
Male	112 (46.5%)	4 (44.4%)	
Age (years)	44.07 ± 14.67	45.25 ± 18.99	.829
BMI (kg/m^2^)			
<18.5	40 (16.6%)	2 (22.2%)	.462
18.5–23.9	141 (58.5%)	2 (22.2%)	
24–27.9	44 (18.3%)	4 (44.5%)	
≥28.0	16 (6.6%)	1 (11.1%)	
Hypertension			
No	192 (79.7%)	6 (66.7%)	.979
Yes	49 (20.3%)	3 (33.3%)	
Diabetes			
No	212 (88.0%)	7 (77.8%)	.565
Yes	29 (12.0%)	2 (22.2%)	
CRD			
No	230 (95.4%)	5 (55.6%)	<.001
Yes	11 (4.6%)	4 (44.4%)	
Coronary heart disease			
No	216 (89.6%)	7 (77.8%)	.497
Yes	25 (10.4%)	2 (22.2%)	
Tumor size	19.56 ± 7.82	23.75 ± 10.35	.154
Pathology			
NF	181 (75.1%)	6 (66.7%)	
ACTH	19 (7.9%)	1 (11.1%)	
GH	38 (15.8%)	2 (22.2%)	
PRL	3 (1.2%)	0 (0.00%)	
Consistency of the adenoma			
Tenacious	119 (49.4%)	5 (55.6%)	.383
Soft	122 (50.6%)	4 (44.4%)	.451
Knosp Grade			.864
0	42	1	
1–2	145	5	
3–4	54	3	
Degree of tumor resection			.011
GTR	208	6	
STR	27	3	
PTR	6	0	
Intraoperative CSF leak			.021
No	173 (71.8%)	2 (22.2%)	
Yes	68 (28.2%)	7 (77.8%)	
Sella reconstruction methods			<.001
synthetic dura+biological glue	166 (68.9%)	3 (33.3%)	
abdominal fat+ dura+free graft	28 (11.6%)	6 (66.7%)	
abdominal fat+ dure+VNSF	47 (19.5%)	0 (0%)	
lumbar subarachnoid drain		0.286	
No	226 (93.8%)	7 (77.8%)	
Yes	15 (6.2%)	2 (22.2%)	

### 3.4 Multivariate analysis

Multivariate analysis was performed using variables that were significantly correlated with intraoperative CSF leakage. However, in the multivariate analysis (Table 2), only the tumor size (*P* <.001; OR, 1.229) was independently associated with an increased intraoperative CSF leakage rate. Similarly, multivariate analysis was performed using variables that significantly correlated with postoperative CSF leakage (Table 4). The results indicated that CRD (*P* < .001; OR, 57.500) and intraoperative CSF leakage (*P* = .022; OR, 7.707) were independent risk factors for postoperative CSF leakage. In contrast, the use of VNSF + abdominal fat (*P* = .039; OR, 0.107) was an independent protective factor for postoperative CSF leakage.

**Table 4:** Multivariate analysis of impact of clinical characteristics upon postoperative CSF leak.

Variable	OR	95% CI for OR	*P* value
CRD	57.500	8.031–411.682	<.001
Intraoperative CSF leak	7.707	1.336–44.455	.022
VNSF+ abdominal fat graft	0.107	0.013–0.894	.039

## 4 Discussion

EETS has continued to improve owing to recent advances in equipment, hemostatic agents, and closure techniques. The incidence of CSF leakage has been reduced to between 0.5% to 12% following EETS.^[1,7–9]^ However, there are still some factors unrelated to surgical skills that could impact the rates of intra- and postoperative CSF leakages, and these have remained poorly defined. Our data suggested that certain factors may predict a greater likelihood of CSF leakage following EETS both intra- and postoperatively.

In most studies of pituitary adenomas and CSF leakages after transnasal surgery, researchers have found that postoperative CSF leakage occurred in most patients with intraoperative CSF leakage.^[3,4,10]^ Thus, intraoperative CSF leakage seems to be an important risk factor for postoperative CSF leakage. Our results confirmed these findings. In our study, nine patients had postoperative CSF leakages. Among them, seven had intraoperative CSF leakages. The final multivariate regression analysis showed that intraoperative CSF leakage was an independent risk factor for postoperative CSF leakage (OR, 7.707; *P* = .022).

Based on these results, to reduce the rate of postoperative CSF leakage, it is important to prevent intraoperative CSF leakage. Moreover, understanding the risk factors of intraoperative CSF leakage is beneficial in the management and prevention of postoperative CSF leakage. It is obvious that all the factors that can induce diaphragma sellae rupture can lead to CSF leakage. Thus, the factors impacting the rupture of the diaphragma sellae can also lead to intraoperative CSF leakage. We observed the significant difference in the tumor size between those who had an intraoperative CSF rhinorrhea and those who did not (16.84 ± 6.16 vs 26.20 ± 7.83; *P* < .001). Zhou et al^[4]^ also reported a high incidence of intraoperative CSF leakage following a large pituitary adenoma resection. Their final multi-factor regression analysis demonstrated that it was an independent risk factor for intraoperative CSF rhinorrhea. Tumor size as a risk factor for CSF leakage during surgery may be due to suprasellar extension in large-sized tumors, which can lead to a rather thin diaphragm sellae. Therefore, gentle manipulation is necessary for large pituitary adenomas to avoid rupture of a rather thin diaphragm sellae during surgery. Removing the tumor in the following order—rear, two sides, and front—may avoid dropping the diaphragma sellae too early and too fast (which may lead to its rupture and consequently CSF leakage).

Our results revealed that CRD increased the risk of postoperative CSF leakage (OR, 57.5; *P* < .01). To our knowledge, this has never been reported in the literature to date. This result may be related to the increased intracranial pressure during the acute onset of CRD. The increased intrathoracic pressure leading to decreased blood flow in the jugular vein, resulting in increased intracranial pressure, may cause herniation of the repaired material from the sellar base and thus, CSF leakage. Two other studies^[11,12]^ have shown that an increased intracranial pressure plays an important role in the occurrence of postoperative CSF leakage or spontaneous CSF leakage. Hanba et al^[12]^ discovered that patients with asthma are more likely to have CSF leakage than patients without asthma after transnasal surgery. Furthermore, the rates of postoperative CSF leakage in these two groups were 4.7% and 2.7%, respectively. Moreover, Fleischman et al^[11]^ found that patients with obstructive sleep apnea were more likely to develop spontaneous CSF leakage.

Previous studies^[3,13]^ have already reported that BMI was a risk factor for postoperative CSF leakage following EETS. However, the incidence of CSF leakage did not correlate with BMI in our series. Dlouhy et al^[13]^ and Karnezis et al^[3]^ both indicated that BMI was a risk factor for postoperative CSF leakage. However, the average BMI of the CSF leakage group compared with that of the non-CSF leakage group in these two studies was 33.1:30.3 kg/m^2^ and 39.2:32.9 kg/m^2^, respectively. In both studies, their patients’ average BMI was greater than 30 kg/m^2^. In our study, the average BMI was 21.80/21.66 kg/m^2^, which was lower than those in previous studies. We think it is possible that the average BMI in our patients was not elevated enough to lead to a spontaneous increase in intracranial pressure that results in postoperative CSF rhinorrhea. On the other hand, it is likely associated with the low variance of the evaluated sample in our study, which may also be one of the reasons that lead to our different conclusions .

Some studies have suggested that a VNFP can reduce the incidence of postoperative CSF leakage to less than 6%.^[14–16]^ Currently, vascularized mucosal flaps has become a recognized method for sellar base reconstruction. The incidence of postoperative CSF leakage in our patients was significantly reduced by the use of VNFP and abdominal fascial graft. The blood supply of the VNFPs was mainly derived from the septal artery, which is rich in blood flow. Thus, the survival rate was higher than those in other non-vascular mucosal flaps.^[14]^ El-sayed et al^[14]^ also pointed out that the atrophy of the mucosa with a VNFP was smaller than that with a non-vascular mucosal flap, which was not enough to detach the mucosal flap from the sellar base.

Whether the use of lumbar subarachnoid drains reduces the rates of postoperative CSF rhinorrhea remains controversial.^[17,18]^ Some researchers noted that the use of intraoperative lumbar subarachnoid drainage did not significantly reduce the postoperative CSF leakage rate.^[18,19]^ However, Stokken et al^[20]^ indicated that in patients with a high-flow intraoperative CSF leakage, the use of a lumbar subarachnoid drain can immediately reduce the risk of postoperative CSF leakage. Conversely, our study found that such use did not significantly reduce the risk of postoperative CSF leakage. Moreover, using a lumbar subarachnoid drain can easily lead to intracranial infection^[18]^ and prolonged hospitalization.^[19]^ Thus, careful consideration is required when using a lumbar subarachnoid drain intraoperatively to prevent postoperative CSF leakage.

In present study, six of nine patients with postoperative CSF leakage were cured with lumbar drainage treatment, the effective rate of lumbar drainage for postoperative cerebrospinal fluid leakage was about 67%. For the treatment of postoperative CSF leakage, the effect of lumbar drains is obvious. Lumbar drains has been indicated to decrease the pressure, which could lower the chance of persistent CSF fistula.^[20]^ However, for the patients with postoperative CSF leakage, four of them developed intracranial infection, the rates of intracranial infection after postoperative CSF leakage was about 44.4%, which was similar to that Michael E. Ivan and his colleagues reported in their study.^[21]^ The leakage of CSF to the sinuses increases exposure to the normal flora in the nasopharynx, then leads to infection,^[22]^ so the use of antibiotics should be more aggressive in patients with postoperative cerebrospinal fluid leakage.

### 4.1 Study limitations

This study has some limitations. First of all, this is a retrospective analysis, we need to design a randomized controlled clinical trial in the future to further verify the reliability of our results in present study. The other limitation is that we did not compare and analyze the cerebrospinal fluid leakage in other types of tumors, such as craniopharyngioma, tuberculum sella meningioma, rathke cyst, which is also the focus of our future research.

## 5 Conclusion

During nasal transsphenoidal endoscopic resection of pituitary adenomas, patients with a large tumor should be strictly monitored for intraoperative CSF leakage. In the presence of intraoperative CSF leakage, the sellar base defects should be carefully repaired to avoid postoperative CSF leakage. The sella constructed by abdnominal fat + VNFP may lower the rate of postoperative CSF leakage. As such, a VNSF should be created ahead of time for these patients. For patients with CRD, postoperative management must be provided with caution. Respiratory management is necessary to reduce inflammatory reactions in the postoperative period. These measures can effectively reduce the incidence of postoperative CSF leakage in patients who are preparing for transnasal sphenoidal endoscopic surgery for pituitary adenomas.

## Author contributions

YP conceived and designed the study. MW and YC performed the statistical analyses. YJ, MW, and YC contributed to data collection and interpretation, as well as drafting and critically revising the manuscript. All authors approved the final version of the manuscript and are accountable for the accuracy and integrity of all aspects of the study.

**Conceptualization:** Ming Wang.

**Data curation:** Ming Wang, Yang Cai, Yugang Jiang, Yong Peng.

**Formal analysis:** Yugang Jiang, Yong Peng.

**Investigation:** Yong Peng.

**Methodology:** Ming Wang, Yong Peng.

**Project administration:** Ming Wang.

**Resources:** Ming Wang, Yang Cai, Yong Peng.

**Software:** Ming Wang, Yang Cai, Yong Peng.

**Validation:** Ming Wang, Yugang Jiang, Yong Peng.

**Writing – original draft:** Ming Wang.

**Writing – review & editing:** Ming Wang, Yong Peng.
